# LncRNA MIAT inhibits osteoblast differentiation and function in rheumatoid arthritis via let-7i-5p/ CKIP-1 axis

**DOI:** 10.1186/s13075-026-03798-7

**Published:** 2026-03-20

**Authors:** Hanxiao Zhao, Jun Shu, Fenghua Yu, Cheng Lu, Li Li, Ning Zhao, Aiping Lu, Xiaojuan He

**Affiliations:** 1 Institute of Basic Research in Clinical Medicine, China Academy of Chinese Medical Sciences, Beijing, 100700 China; 2 School of Traditional Chinese Medicine, Naval Medical University, Shanghai, China; 3 Institute of Clinical Medical Science, China-Japan Friendship Hospital, Beijing, 10029 China; 4 Law Sau Fai Institute for Advancing Translational Medicine in Bone and Joint Diseases, School of Chinese Medicine, Hong Kong Baptist University, Hong Kong, China; 5 Guangdong-Hong Kong-Macau Joint Lab on Chinese Medicine and Immune Disease Research, Guangzhou, China

**Keywords:** Rheumatoid arthritis, Osteoblast, lncRNA MIAT, let-7i-5p, CKIP-1

## Abstract

**Background:**

The dysfunction of osteoblasts has been identified as a pivotal contributor to bone erosion in rheumatoid arthritis (RA). Emerging evidence shows long non-coding RNAs (lncRNAs) as important regulators in various pathological processes, including RA. However, the specific regulatory mechanisms of lncRNAs in osteoblast dysfunction of RA remain poorly understood and worth further investigation.

**Methods:**

Collagen induced arthritis (CIA) rat model was established, tarsal tissue was isolated for sequencing of lncRNAs. The differentially expressed lncRNA was screened and verified in vivo and in vitro. The target microRNA of lncRNA and the target protein of microRNA were predicted by bioinformatics analysis and validated. The biology function of the lncRNA and microRNA was further investigated by knockdown or overexpression experiment in murine osteoblastic cell MC3T3-E1.

**Results:**

LncRNA MIAT was highly expressed in tarsal tissue and serum of CIA rat as well as TNF-α-induced osteoblasts, and its high expression was negatively correlated with the expression of bone formation marker osteocalcin. Functionally, knockdown of lncRNA MIAT promoted the proliferation and mineralization of osteoblasts. Mechanistically, lncRNA MIAT directly bound to let-7i-5p and exerted a negative regulatory influence on its expression. CKIP-1 was a target of let-7i-5p. Overexpression of let-7i-5p promoted osteoblast proliferation and mineralization by targeting CKIP-1.

**Conclusions:**

Taken together, this study demonstrated that lncRNA MIAT was involved in the regulation of osteoblast proliferation and mineralization through the lncRNA MIAT/let-7i-5p/CKIP-1 axis in RA.

**Supplementary Information:**

The online version contains supplementary material available at 10.1186/s13075-026-03798-7.

## Introduction

Rheumatoid arthritis (RA) is a common chronic autoimmune disease, characterized by chronic inflammation and progressive bone erosion [1, 2]. More than 10% of RA patients appear bone erosions within 8 weeks of disease onset, and as many as 60% develop erosions after 1 year [3]. Current treatment strategies for RA are mainly focused on the suppression of overactive immune system and symptomatic treatment, lacking targeted treatment for bone erosion [4]. Although the newly treatment strategy such as TNF-α inhibitors, IL-6 inhibitors and JAK inhibitors can partially inhibit the progress of bone erosion, it does not significantly reduce the incidence of fracture [5, 6]. This indicates that there are still unknown mechanisms for the bone erosion in RA. Therefore, deeper understanding the molecular mechanism may pave the way for the prevention and blocking of bone damage in RA.

Bone erosion in RA mainly lies in excessive osteoclast-mediated bone absorption and insufficient osteoblast-mediated bone formation [5, 7]. Previous studies on bone erosion of RA mostly focused on osteoclasts, but little attention was paid to osteoblasts. Actually, recent studies have demonstrated that the maturation and mineralization of osteoblast is compromised at sites of focal bone erosion in RA. However, the exact molecular mechanism is not clear [8]. Osteoblasts are derived from mesenchymal stem cells (MSCs) in bone marrow and regulated by various factors during differentiation [7]. For example, WNT and bone morphogenetic protein (BMP) pathway related-proteins can inhibit the differentiation of MSCs into adipocytes and the apoptosis of osteoblast precursors, and promote the differentiation of osteoblasts [9, 10].

Long noncoding RNAs are RNAs greater than 200 nucleotides long that do not have the ability to encode proteins [11]. These lncRNAs can regulate gene expression through a variety of mechanisms [12]. LncRNAs or mRNAs can bind to microRNAs (miRNAs) seed sequences via miRNA recognition elements (MREs). In this way, lncRNAs can promote the expression of miRNA-targeted mRNAs by specifically sponging on corresponding miRNAs. By forming the above competitive endogenous RNAs (ceRNAs) network, lncRNAs can regulate various biological processes [13]. LncRNAs have been detected as dysregulated in patients with a variety of diseases, and are widely involved in the pathogenesis of diseases, such as cancer, lung, cardiovascular and autoimmune diseases [14–17]. Recent studies have shown that many lncRNAs are abnormally expressed in patients with RA and play an important role in the pathogenesis of RA [18–20]. For example, the average level of serum lncRNA GAS5 in RA patients decreased and was negatively correlated with common rheumatic indicators [21]. Another study showed that lncRNA MEG3, MALAT1 and NEAT1 in RA patients can predict the clinical parameters of RA [22]. As the target genes of lncRNAs, miRNAs are regarded as key regulatory factors involved in bone repair and regeneration [23, 24]. Sun Y et al. found that lncRNA OIP5-AS1 could promote the activation of the Wnt/β-catenin pathway in fibroblast-like synoviocytes by regulating the miR-410-3p/Wnt7b signaling axis, thereby participating in the occurrence and development of RA [25]. With the deepening of the research, growing evidences showed that lncRNAs act an irreplaceable role in the formation and development of osteoblasts [26, 27]. For instance, lncRNA XIST participated in the proliferation and differentiation of osteoblasts by regulating miR-203-3p [28]. LncRNA MCF2L-AS1 positively regulated the expression of Runx2 and promoted the osteogenic differentiation of human bone marrow MSCs by sponging miR-33a [29]. As one of the earliest known miRNAs, the let-7 family members let-7c, let-7f and let-7e-5p all have the function of promoting bone formation differentiation and bone formation [30, 31]. For example, let-7e-5p regulated the differentiation of osteoblasts through the JAK2/STAT5 pathway by inhibiting SOCS1 [31]. However, the effect of lncRNA on osteoblasts of RA is still obscure and needs to be further studied.

Therefore, the purpose of this study was to explore the role and mechanism of lncRNA as an important regulator in the differentiation and function of osteoblasts as well as bone erosion in RA.

## Materials and methods

### Animals

Male Sprague Dawley rats aged 6–8 weeks were from Beijing Vital River Laboratory (Beijing, China), and were kept in specific pathogen-free facilities. Before the beginning of the experiment, the rats were randomly fed with sterilized food and water for one week. This research plan was approved by the research ethics committee of Institute of Basic Theory for Chinese Medicine, China Academy of Chinese Medical Sciences (No.2019-050).

### CIA model induction

After adaptive feeding, bovine collagen type II and incomplete Freund’s adjuvant (Chondrex, Redmond, WA, USA) were mixed in a ratio of 1:1, completely emulsified and injected 200 µl into the tail of each rat. On the 7th day after the first immunization, 100 µl emulsified mixture was injected again to immunize the rats [32, 33].

The rats were observed every 2 days and assigned an arthritis index score on a scale of 0 to 4: 0 = no edema or swelling, 1 = mild edema and erythema limited to the foot and/or ankle, 2 = mild edema and erythema from the ankle to the tarsus, 3 = moderate edema and erythema from the ankle to the tarsus, and 4 = severe edema and erythema. The sum of the scores of the two hind limbs was the final score for each rat, which could be as high as 8 points.

### Histological analysis

The ankle joints of rats were fixed in 10% phosphate-buffered formalin for 3 days and then decalcified in 10% EDTA (pH = 7.2). Section (4 μm) were stained with hematoxylin and eosin (H&E) for histological assessment, and the histological characteristics were scored on a scale of 0–4 using the criteria described in a previous study [33].

### Transcriptome of the tarsal joints

Three tarsal joints were extracted from the normal group and the model group respectively at day 64. After removing the skin, muscle, tendon and ligament, the bone tissue were obtained to extract total RNA. After the total RNA was identified and quantified, the cDNA libraries were established and sequenced. SOAPnuke (v1.5.2) was used to filter the sequencing data. HISAT2 (v2.0.4) software was then used to map the filtered data to the reference genome. Bowtie2 (v2.2.5) was used to compare the data with the reference coding gene set, and RSEM (v1.2.12) was used to calculate the gene expression level.

### DELs screening

DESeq2 (v1.4.5) was used for differential expression analysis. LncRNAs with |log FC| >1.0 and *P* value < 0.05 were selected and named DELs. The target gene miRNAs of DELs were predicted by lncBase V3 and miRcode.

### RT-qPCR

RNA (2 µg per reaction) was reverse transcribed to cDNA using TRUEscript RT MasterMix (Aibosen Biotechnologies Co., Ltd., Beijing, China). qPCR was performed with 2× SYBR Green qPCR Master Mix (Biomake, Houston, TX, USA). PCR was initiated at 95 °C for 5 min, and the cycling conditions were 95 °C for 10 s, 60 °C for 15 s and 75 °C 15 s (40 cycles). Samples were analyzed in triplicate and normalized by subtraction of the sample mean Ct value from the mean Ct value of the housekeeping gene GAPDH.

### Cell culture

Murine osteoblastic cell MC3T3-E1 was cultured in α-MEM medium containing 10% fetal bovine serum (FBS) and 100 U/ml penicillin and streptomycin. Cells were inoculated in 25 cm^2^ cell culture flasks and incubated in an incubator under the culture temperature of 37 °C, containing 5% CO_2_ and 95% relative humidity. When the confluence of the cells reached 80–90%, the cells were digested with trypsin for subsequent experiments.

### Dual-luciferase reporter assay

The sequence fragment of MIAT embracing the potential binding sites of let-7i-5p and matched mutant was synthesized and embedded into vector (pSI-Check2 vector) named as MIAT-w, MIAT-m, respectively. Subsequently, 293T cells were co-transfected with above vectors along with let-7i-5p or miR-NC with Lipofectamine 3000 (Thermo Fisher Scientific, Waltham, MA USA) and propagated in the medium. After 48 h, luciferase activity was analyzed by dual-luciferase reporter assay kit (Promega, Madison, USA) as instructed by the manufacturer.

### Cell transfection

The small interfering RNA targeting MIAT (MIAT-siRNA), let-7i-5p mimics, the negative control groups (NC-siRNA, NC mimics), were designed by the Tsingke Biotechnology (Beijing, China). MC3T3-E1 cells were inoculated in 6-well plates, when the degree of confluence reached 50%–60%, 50 nM MIAT-siRNA, let-7i-5p mimic, NC-siRNA or NC mimics were transfected into the cells using LabFect RNAi Transfection (LABLEAD, Beijing, China). Six hours after transfection, fresh culture medium containing 10% FBS was replaced to maintain normal growth of cells. Follow-up experiments were conducted 24 h after transfection.

### CCK-8 assay

MC3T3-E1 cells were seeded into 96-well plates at a density of 4 × 10^3^ cells/well. To detect the cell viability, 10 µL of CCK-8 was added into each well at 24 h, 48 h. After the mixture was fully mixed, the cells were placed in a cell incubator in the dark and incubated for 2 h. The absorbance values of each group of cells were detected by enzyme plate analyzer, and the cell proliferation capacity was calculated.

### ALP activity assay and alizarin red staining

MC3T3-E1 cells were inoculated in 6-well plates, induced with OriCell MUXMT-90,021 osteogenic induction medium. On the 7th day after induction, the medium was removed from the culture plates and submitted to detect ALP activity. On the 14th day after induction, ARS staining was used to detect the formation of mineralized nodules in MC3T3-E1 cells.

### Statistical analysis

GraphPad Prism v.6 (GraphPad Software, San Diego, CA, USA) was used for statistical analyses. Measurement data including RT-qPCR were analyzed by ANOVA, followed by Bonferroni’s multiple comparison test. *P* < 0.05 was identified significant.

## Results

### Analysis of differently expressed lncRNAs in tarsal tissue of CIA rats

**Fig. 1:**
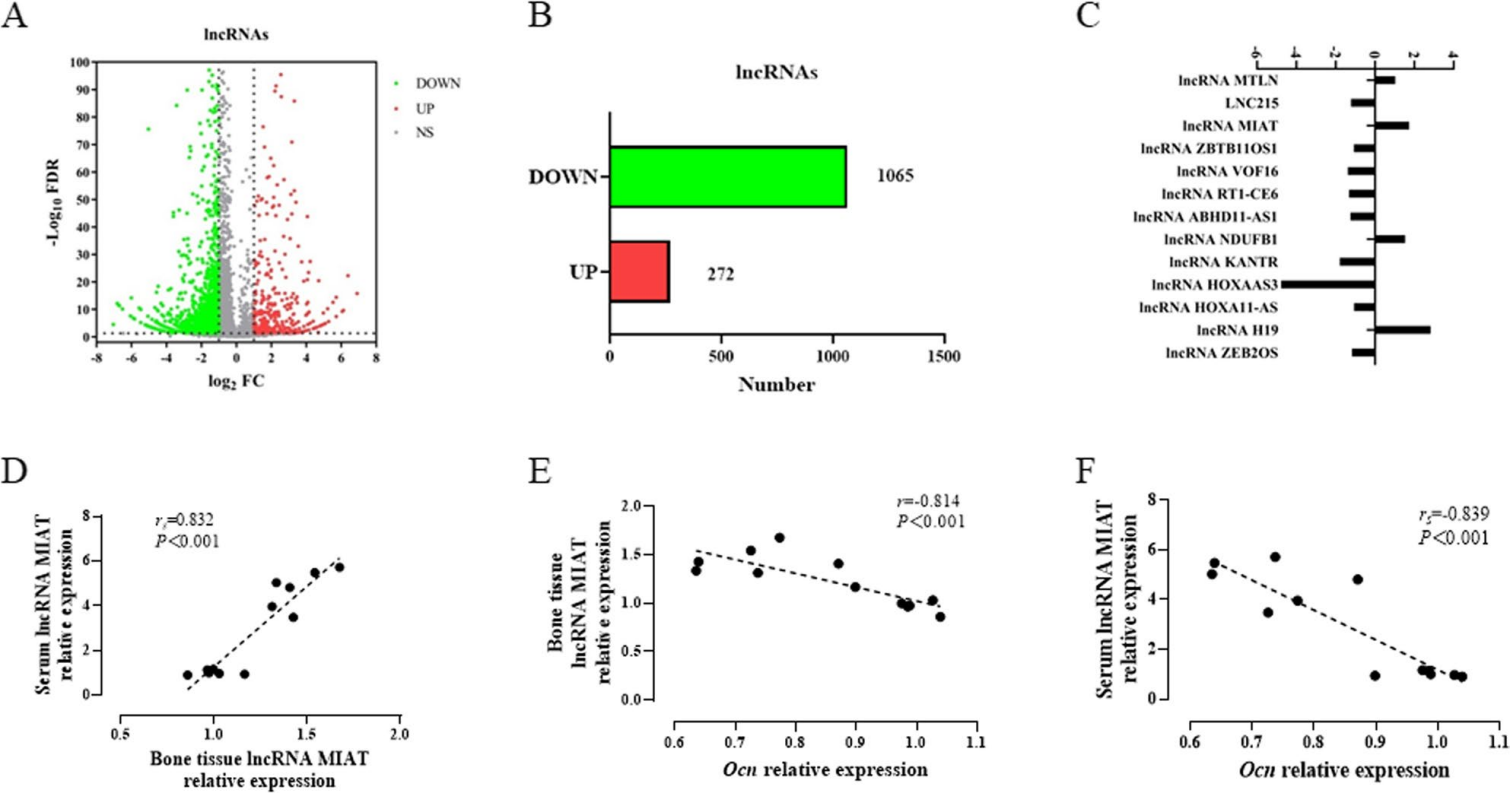
Identification and verification of DELs in tarsal joints of CIA. **A** Volcano plot of lncRNAs. **B** The number of lncRNAs. **C** Fold change of lncRNAs. **D** Correlation analysis between serum lncRNA MIAT and intra-osseous lncRNA MIAT. **E** Correlation analysis between serum lncRNA MIAT level and intra-osseous Ocn level. **F** Correlation analysis between intra-osseous lncRNA MIAT level and intra-osseous Ocn level

In order to find lncRNAs that may be involved in the bone erosion in RA, we established CIA rats and found remarkably bone erosion at day 64 after the first immunization (Supplementary Fig. 1). Then, we collected tarsal tissue for transcriptome analysis. The volcano plot of lncRNAs was shown (Fig. 1A). Compared with the normal group, a total of 1337 lncRNAs were differentially expressed in the model group, among which 1065 lncRNAs were down-regulated and 272 lncRNAs were up-regulated (Fig. 1B). Then, a total of 13 lncRNAs annotated in NCBI were selected from 1337 DELs (Fig. 1C). Four lncRNAs with up-regulated expression were further detected by RT-qPCR. We noted that lncRNA MIAT expression was also significantly increased in the serum of CIA rats than in normal group (Supplementary Fig. 2). Moreover, the serum lncRNA MIAT level was positively correlated with the intra-osseous lncRNA MIAT, whereas negatively correlated with the intra-osseous osteocalcin (*Ocn)* level, a marker of bone formation. Similarly, the intra-osseous lncRNA MIAT level was also negatively correlated with the intra-osseous *Ocn* level (Fig. 1D, E and F). These results suggested that lncRNA MIAT might be related to the reduction of bone formation in RA. Hence, lncRNA MIAT was selected for the subsequent experiments.

### Expression levels of lncRNA MIAT in CIA rats and osteoblasts

**Fig. 2:**
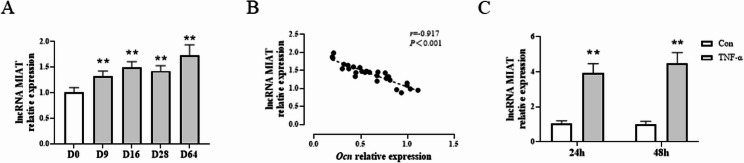
Expression levels of lncRNA MIAT in CIA rats and MC3T3-E1 cells. **A** RT-qPCR analysis of lncRNA MIAT levels at different times in tarsal tissue of CIA rats. **B** Correlation analysis between intra-osseous lncRNA MIAT level and intra-osseous Ocn mRNA level. **C** RT-qPCR analysis of lncRNA MIAT levels in TNF-α (25ng/mL) induced-MC3T3-E1 cells

Then, the expression changes of lncRNA MIAT in tarsal tissue of CIA rats at different time were further detected by RT-qPCR, and the correlation between the expression level of lncRNA MIAT in tarsal tissue of rats and the expression level of *Ocn* was analyzed. RT-qPCR results showed that compared with day0 (D0), the expression level of lncRNA MIAT in tarsal tissue of rats in the model group began to increase at D9 and continued to be high in the advanced stage (*P* < 0.01) (Fig. 2A). Correlation analysis showed that the expression level of lncRNA MIAT in tarsal tissues was negatively correlated with the expression level of *Ocn* in tarsal tissues (*P* < 0.01) (Fig. 2B).

As lncRNA MIAT is highly conservative in mammals [34], we then performed cell experiments in murine osteoblastic cell MC3T3-E1 to further investigate its mechanism of action. TNF-α induction was performed on MC3T3-E1 cells for 24–48 h. RT-qPCR results showed that compared with control group, lncRNA MIAT expression in MC3T3-E1 cells was remarkably up-regulated after the induction of TNF-α (*P* < 0.01) (Fig. 2C). This suggested that TNF-α could promote the expression of lncRNA MIAT in osteoblasts.

### LncRNA MIAT regulated the proliferation and mineralization of osteoblasts

**Fig. 3:**
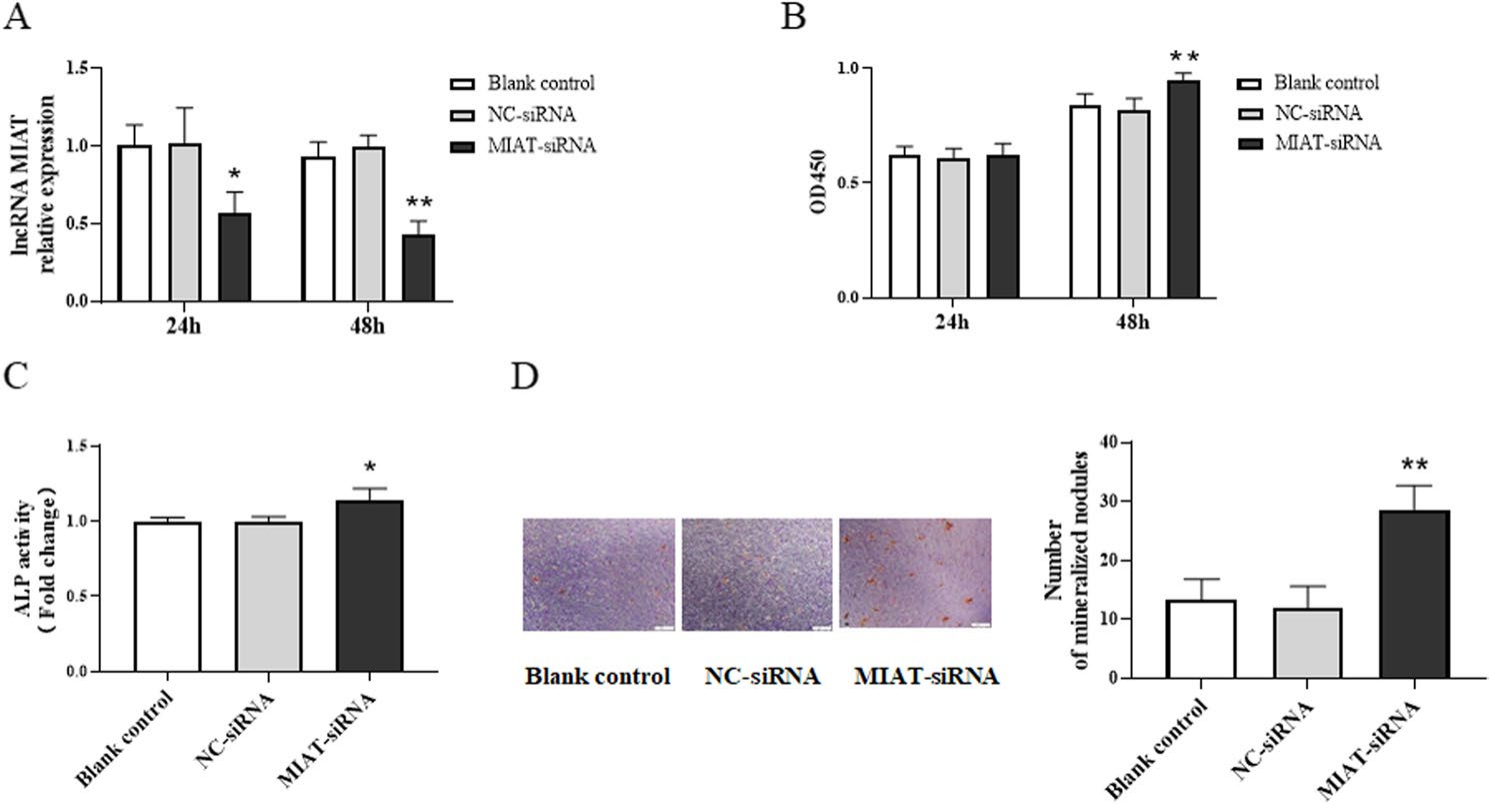
LncRNA MIAT regulates the proliferation and mineralization of MC3T3-E1 cells. **A** RT-qPCR analysis lncRNA MIAT levels in MC3T3-E1 cells after transfection of MIAT-siRNA. **B** CCK-8 analysis of the proliferation of MC3T3-E1 cells after transfection of MIAT-siRNA. **C** ALP activity analysis of MC3T3-E1 cells after transfection of MIAT-siRNA. **D** Alizarin red staining analysis of MC3T3-E1 cells after transfection of MIAT-siRNA. Magnification: 40×

To further explore the role of lncRNA MIAT in osteoblasts, we performed loss-of-function assay by using MIAT siRNA. RT-qPCR results verified the expression level of lncRNA MIAT was significantly down-regulated 24 h and 48 h after transfection with MIAT-siRNA (*P* < 0.05) (Fig. 3A). Then, the CCK-8 assay was used to assess the proliferation of osteoblasts. The results showed that the proliferation of MC3T3-E1 cells in MIAT-siRNA group was remarkably promoted compared to the control group and NC-siRNA group at 48 h after transfection (*P* < 0.01) (Fig. 3B). Then, MC3T3-E1 cells were treated by osteogenic induction medium, the ALP activity was detected and the results showed the activity of ALP was significantly increased in MIAT-siRNA group when compared with the control group and NC-siRNA group (*P* < 0.05) (Fig. 3C). After 14d of osteogenic induction, alizarin red staining was performed to detect the calcified nodules of osteoblasts. The results showed that the MIAT-siRNA group had more calcified nodules than the control group and NC-siRNA group (Fig. 3D). These results indicated that inhibiting the expression of lncRNA MIAT could promote the proliferation and mineralization of osteoblasts.

### Verification of miRNAs that can bind to lncRNA MIAT

**Fig. 4:**
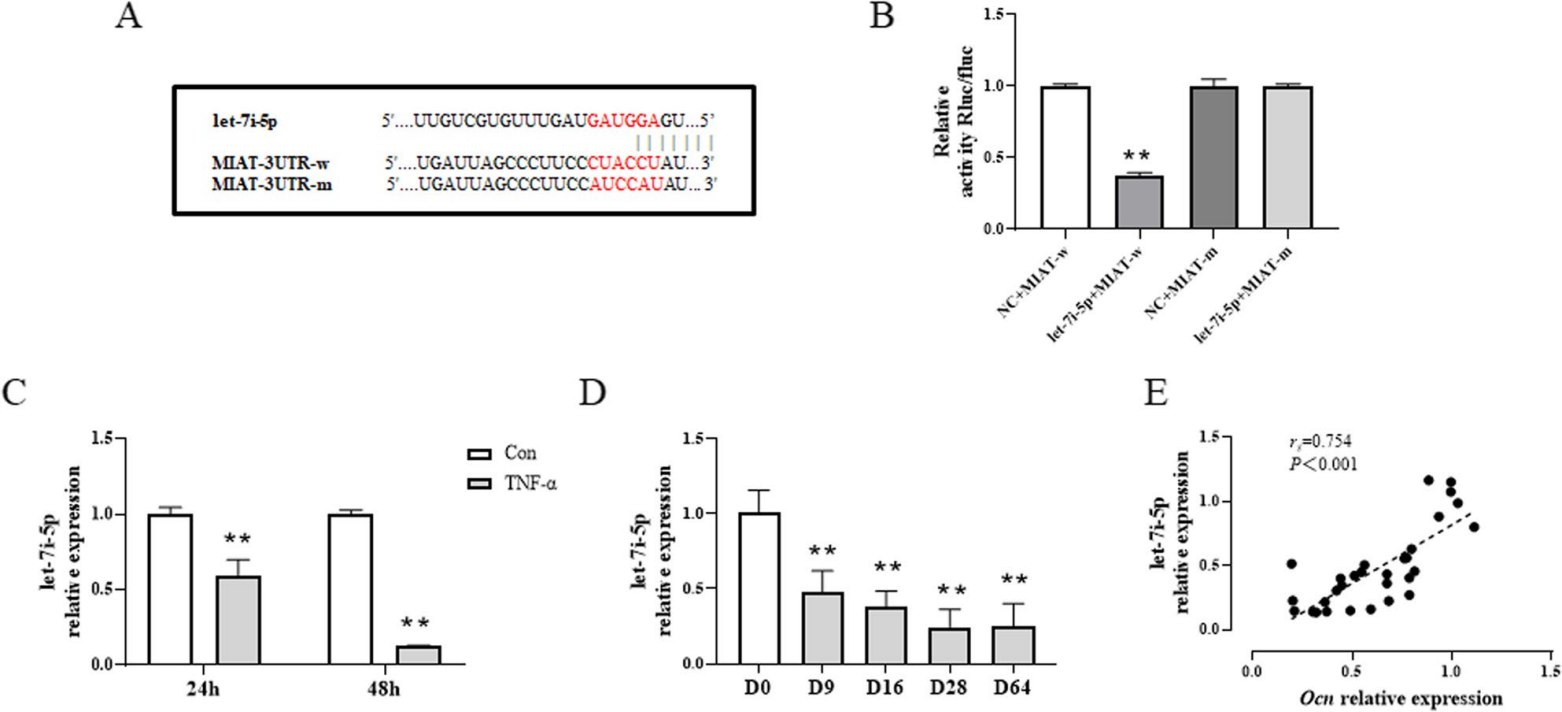
LncRNA MIAT targets let-7i-5p. **A** Sequence of predicted binding sites. **B** Validation of dual-luciferase reporter assay. **C** RT-qPCR analysis of let-7i-5p levels in TNF-α (25ng/mL) induced-MC3T3-E1 cells. **D** RT-qPCR analysis of let-7i-5p levels at different times in tarsal tissue of CIA rats. **E**Correlation analysis between let-7i-5p level and Ocn level in CIA rats

Accumulating studies have reported that lncRNAs could function as competing endogenous RNAs (ceRNAs) to sponge miRNA targets. To screen and verify the miRNA targets that can interact with lncRNA MIAT, we used a bioinformatics analysis and predicted that let-7i-5p, which belongs to let-7 family members, might be one of target miRNAs of lncRNA MIAT. Further, a dual-luciferase report gene assay was performed. As expected, the let-7i-5p mimics inhibited the luciferase activity in the wild-type lncRNA MIAT vector, which verified the binding of lncRNA MIAT to let-7i-5p (Fig. 4A and B). Then, we tested the level of let-7i-5p by RT-qPCR in MC3T3-E1 cells upon induction with TNF-α. The results demonstrated that the level of let-7i-5p was significantly down-regulated in TNF-α-induced MC3T3-E1 cells compared to the control (*P* < 0.01) (Fig. 4C). Meantime, let-7i-5p level was obviously lower in tarsal tissue of CIA rats. The correlation analysis indicated that the level of let-7i-5p was positively correlated with the level of *Ocn* in the tarsal tissue of CIA rats (*P* < 0.01) (Fig. 4D and E).

**Fig. 5:**
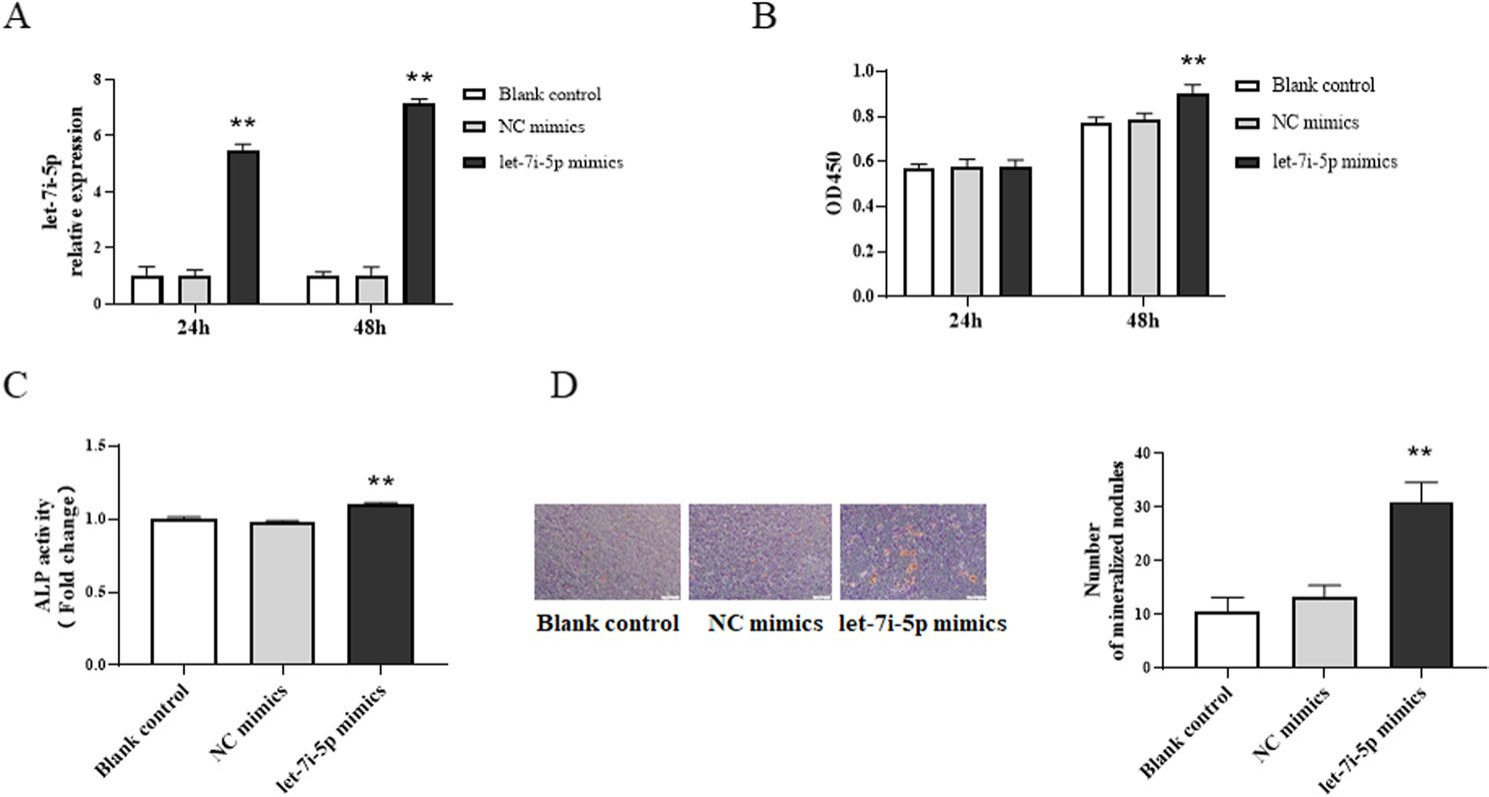
let-7i-5p regulates the proliferation and mineralization of MC3T3-E1 cells. **A** RT-qPCR analysis of let-7i-5p level in MC3T3E1 cells after transfection of let-7i-5p mimics. **B** CCK-8 analysis of the proliferation of MC3T3-E1 cells after transfection of let-7i-5p mimics. **C** ALP activity analysis of MC3T3-E1 cells after transfection of let-7i-5p mimics. **D** Alizarin red staining of MC3T3-E1 cells after transfection of let-7i-5p mimics. Magnification: 40×

To further explore the role of let-7i-5p in osteoblasts, we performed cell transfection experiments. RT-qPCR showed that the level of let-7i-5p was up-regulated 24 h and 48 h after transfection with let-7i-5p mimic (*P* < 0.01) (Fig. 5A). CCK-8 assay showed that the proliferation of MC3T3-E1 cells in let-7i-5p mimic group was remarkably promoted when compared with the control group (*P* < 0.01) (Fig. 5B). Furthermore, the ALP activity was enhanced and the calcified nodules of MC3T3-E1 cells treated with osteogenic induction medium were also significantly increased in let-7i-5p mimic group compare to the control group (Fig. 5C and D). These results indicated that promoting the expression of let-7i-5p could promote the proliferation and mineralization of osteoblasts.

### Let-7i-5p targeted CKIP-1 in osteoblasts

**Fig. 6:**
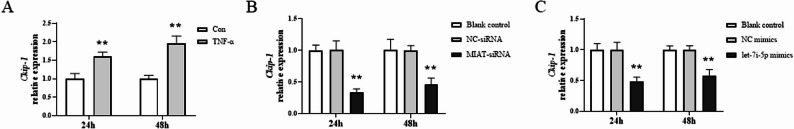
LncRNA MIAT and let-7i-5p influence the expression of *Ckip-1* in MC3T3-E1 cells. **A** RT-qPCR analysis of Ckip-1 mRNA level in TNF-α (25ng/mL) induced-MC3T3-E1 cells. **B** RT-qPCR analysis of Ckip-1 mRNA level after transfection of MIAT-siRNA. **C** RT-qPCR analysis of Ckip-1 mRNA level after transfection of let-7i-5p mimics

**Fig. 7:**
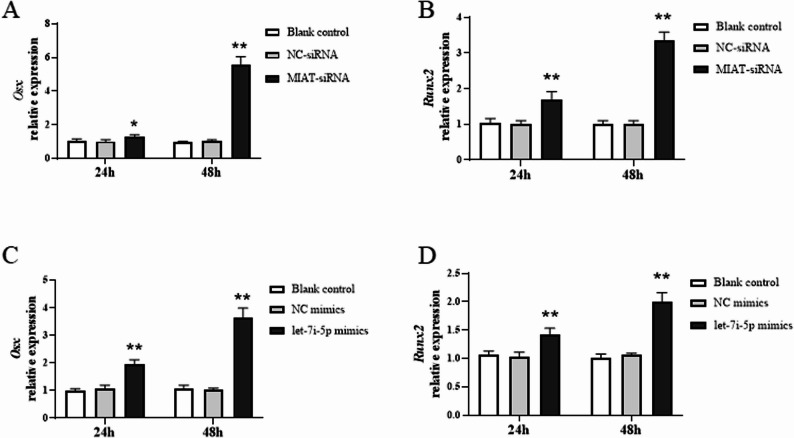
LncRNA MIAT and let-7i-5p influence the expression of Osx and Runx2 in MC3T3E-1 cells. **A** RT-qPCR analysis of Osx mRNA level after transfection of MIAT-siRNA. **B** RT-qPCR analysis of Runx2 mRNA level after transfection of MIAT-siRNA. **C** RT-qPCR analysis of Osx mRNA level after transfection of let-7i-5p mimics. **D** RT-qPCR analysis of Runx2 mRNA levels after transfection of let-7i-5p mimics

Casein kinase-2 interacting protein-1 (CKIP-1), also known as pleckstrin homology domain containing protein O1 (PLEKHO1), plays a critical negative regulatory role during the bone morphogenetic process. In our previous studies, we have demonstrated that CKIP-1 was highly expressed in osteoblasts of articular specimens from RA patients and CIA animal model. Genetic deletion or therapeutic inhibition of osteoblastic CKIP-1 could effectively suppress the reduction of bone formation [35]. In addition, previous research has reported CKIP-1 was one of targets of let-7i-5p in osteoblasts. A dual-luciferase report gene assay has performed to verify the interaction of CKIP-1 with let-7i-5p [20]. Based on these points, we further explored the functional role of lncRNA MIAT and let-7i-5p in CKIP-1 regulation of osteoblasts. RT-qPCR results showed that the mRNA expression of *Ckip-1* was markedly up-regulated 24 h and 48 h in TNF-α-induced MC3T3-E1 cells (Fig. 6A). After transfection with MIAT-siRNA, the mRNA expression of *Ckip-1* was remarkably down-regulated (Fig. 6B). The similar result was also found in MC3T3-E1 cells after transfection with let-7i-5p mimic (Fig. 6C). Additionally, the mRNA expression of osteogenic related indicators *Osx* and *Runx2* were significantly up-regulated (*P* < 0.05) in MC3T3-E1 cells after transfection with MIAT-siRNA or let-7i-5p mimic (Fig. 7). These results demonstrated that lncRNA MIAT and let-7i-5p could influence the proliferation and mineralization of osteoblasts through regulating the mRNA expression of *Ckip-1*.

## Discussion

Long non-coding RNA myocardial infarction associated transcription (lncRNA MIAT), also known as retina non-coding RNA2 or Gomafu, is highly conserved in mammals [36]. Previous studies have shown that lncRNA MIAT is a carcinogenic factor in a variety of malignant tumors, and its high expression is related to the clinicopathological characteristics of cancer patients [36, 37]. Recent studies suggested that the expression level of lncRNA MIAT may be closely related to bone-related diseases. Clinical studies have found that the expression of lncRNA MIAT in serum and peripheral blood mononuclear cells of patients with osteoporosis was significantly up-regulated, and the increase of lncRNA MIAT was associated with the development of osteoporosis [38, 39]. Another study found that lncRNA MIAT played an important role in the pathogenesis of femoral head necrosis [40]. In addition, high expression of lncRNA MIAT was also found in the synovium of CIA mice [41]. In the synovial fibroblasts of RA patients, lncRNA MIAT and the inflammatory factor matrix metalloproteinase 9 (MMP9) were found to be highly expressed, and the disease progression was involved through the lncRNA MIAT/miR-204-5p/MMP9 axis [42]. Our study indicated that lncRNA MIAT in tarsal tissue of rats in CIA model group was significantly up-regulated compared with normal group. Dynamic detection results showed that lncRNA MIAT in tarsal tissue of CIA rats began to increase on the 9th day of initial immunization and continued to increase in the advanced stage, and the expression of lncRNA MIAT was correlated with reduced bone formation. Compared with the normal group, the serum lncRNA MIAT in CIA rats was also significantly up-regulated. Serum lncRNA MIAT expression was not only positively correlated with lncRNA MIAT expression in tarsal tissue, but also with decreased bone formation in CIA rats. This suggested the potential of serum lncRNA MIAT as a biological indicator of bone formation changes in RA.

Previous studies have shown that TNF-α is a negative regulator of osteogenesis and can inhibit the proliferation and function of osteoblasts [43]. In addition, the expression of lncRNA MIAT was significantly down-regulated during osteogenic differentiation [39, 44]. Moreover, growing evidences have shown that some inflammatory cytokines including TNF-α can regulate the levels of lncRNAs in RA cells [45, 46]. After TNF-α induction, lncRNA MIAT expression was significantly increased, and down-regulation of lncRNA MIAT expression reversed the negative effect of TNF-α on osteoblast differentiation [44]. In our study, we found that inhibiting lncRNA MIAT expression significantly promoted the proliferation and mineralization of osteoblasts. However, the negative regulation of lncRNA MIAT on osteoblasts still needs more in vivo and in vitro experiments for further verification.

Bioinformatics analysis showed that let-7 family members were the target of lncRNA MIAT. The let-7 family is one of the largest and most conserved miRNA families [47]. Family members are let-7a-1, let-7a-2, let-7a-3, let-7b, let-7c, let-7d, let-7e, let-7f-1, let-7f-2, let-7 g, let-7i, and miR-98 [48]. Previous studies have shown that the let-7 family can promote cell differentiation and inhibit tumor [49].Let-7a and let-7e are associated with early inflammation and apoptosis, while let-7i and let-7f are involved in the regulation of late transcription and apoptosis [50]. Animal studies have shown that the expression levels of let-7c and let-7d in mouse embryos were relatively low, and the expressions of femur were significantly increased after birth. Let-7c and let-7d were also up-regulated during osteogenic differentiation of adipose MSCs. After inhibition of let-7 expression, osteoblast differentiation was also inhibited. These results suggested that let-7 can promote osteogenic differentiation and bone formation of human adipose MSCs [51]. In addition, let-7e-5p mimics promoted osteoblast differentiation by positively regulating the expression levels of osteoblast-related genes (*Runx2*,* Opn*,* Ocn*,* and Osx*), enhancing ALP activity and the formation of mineralized nodules. Let-7e-5p regulated osteogenic differentiation by inhibiting SOCS1. This action leaded to activation of the JAK2/STAT5 signaling pathway, which in turn leaded to up-regulation of insulin-like growth factor 1 (IGF-1)expression [31]. In the process of inhibiting osteogenic differentiation of mouse bone marrow MSCs, dexamethasone (Dex) inhibited the expression of let-7f-5p. Let-7f-5p promoted Dex-inhibited osteoblast differentiation in vitro. Let-5f-7p treatment also prevented Dex-induced bone loss in mice [52]. In rat bone marrow MSCs, let-7f-5p could promote the proliferation and osteogenic differentiation by acting as a positive regulator of tumor necrosis factor receptor 2 [53]. In our study, the direct binding effect between lncRNA MIAT and let-7i-5p was confirmed by the results of double luciferase reporter gene detection. Further study showed that let-7i-5p expression in tarsal tissue of CIA rats was significantly down-regulated, and began to decrease on the 9th day of initial immunization, and continued to decrease in the advanced stage. The expression of let-7i-5p was positively correlated with bone formation. After TNF-α intervention, the expression of let-7i-5p in osteoblasts was also significantly down-regulated, and the up-regulated expression of let-7i-5p could significantly promote the proliferation and mineralization of osteoblasts. These results indicated that let-7i-5p was involved in the regulation of osteoblasts and bone formation.

CKIP-1, a target of let-7i-5p, is closely related to bone formation. Previous studies have confirmed that let-7i-5p might promote osteogenic differentiation and fracture healing by inhibiting *Ckip-1* expression [20]. One study showed that *Ckip-1* knockout partially could offset the symptoms of microgravity-induced osteoporosis in mice [54]. Another result showed that the femur bone volume and bone density of *Ckip-1* knockout mice were significantly higher than those of wild-type mice [55]. In addition, high expression of CKIP-1 may promote Smad1 ubiquitination, inhibit Smad-dependent BMP signaling, and inhibit osteogenic differentiation and mineral deposition of MC3T3-E1 cells during glucocorticoid therapy. Osteoblast targeting *Ckip-1* siRNA therapy also inhibited decreased bone formation in glucocorticoid-induced osteoporosis mice and aging mice [56, 57]. Therefore, CKIP-1 is considered as a potential target for the treatment of osteoporosis [58]. Multiple in vitro experiments have also shown that CKIP-1 is a negative regulator of osteogenesis, and bone marrow MSC derived from *Ckip-1* knockout mice showed greater osteogenic ability [55, 59]. Another study showed that CKIP-1 negatively regulated C10H1T2/5 cell proliferation and osteogenic differentiation through the WNT signaling pathway LRP5 [60]. In addition, knockdown of *Ckip-1* promoted osteogenic differentiation of human dental pulp stem cells through BMP2-Smad1/5 signaling, while overexpression of *Ckip-1* had a negative effect on osteogenic differentiation [61]. The results of RA related studies showed that *Ckip-1* was highly expressed in the knee bone tissue of RA patients. The level of CKIP-1 in ankle osteoblasts of CIA group increased from the early stage of modeling and continued to increase during disease progression. The bone tissue structure and bone mass of CIA mice with *Ckip-1* gene knockout were better than those of wild-type CIA mice. In CIA mice and CIA cynomolgus monkey models, *Ckip-1* siRNA treated animals had better bone tissue structure and higher bone mass [35]. In our study, we found the expression of *Ckip-1* in osteoblasts was also significantly increased after TNF-α intervention. After down-regulating lncRNA MIAT expression, *Ckip-1* mRNA expression in osteoblasts was significantly decreased. After up-regulation of let-7i-5p expression, *Ckip-1* mRNA expression of osteoblasts was also significantly decreased. These results demonstrated that lncRNA MIAT and let-7i-5p could influence the proliferation and mineralization of osteoblasts through regulating the mRNA expression of *Ckip-1*.

In conclusion, lncRNA MIAT level was significantly elevated in serum and tarsal tissue of CIA rats, and its level was closely related to bone formation. LncRNA MIAT as a regulatory factor in RA can participate in the regulation of osteoblast differentiation through lncRNA MIAT/let-7i-5p/CKIP-1 axis. Of course, this experiment has also some limitations. The level of lncRNA MIAT in RA patients has not yet been verified. Moreover, this study only demonstrated the effects of MIAT-siRNA and let-7i-5p mimics on MC3T3-E1 cells, which need to be further verified in RA animal models.

## Supplementary Information

Supplementary Material 1

## Data Availability

The datasets used and/or analysed during the current study are available from the corresponding author on reasonable request.
