# Supplementary material for: LncRNA MIAT inhibits osteoblast differentiation and function in rheumatoid arthritis via let-7i-5p/ CKIP-1 axis
Source: Arthritis Res Ther. 2026 Mar 20;28:98. doi: 10.1186/s13075-026-03798-7 (PMC13126773; doi:10.1186/s13075-026-03798-7)
Supplement: Supplementary file 1 — Supplementary Material 1 [file 13075_2026_3798_MOESM1_ESM.docx]

**Supplementary data**

In the dual-luciferase assay, the sequence of the internal reference gene is as follows:

MIAT-w： CACTGAGAAGCATCTTTGCAGATAAGTATTTAAACTTACCAGCCCATAGCCTACCAGCCTGCCCTCTTCTGGTCTGTGCAGGAAAGTGTAGTATCCTAGAATGCCAAAGTGGGAGGGGAAATGGGTGATGTAGCTCATTCTCTTTTCTACCTCTATGGAAGAAAGAAAGAGCCTGTCCCCATTTTGTGGGGTCCCAGAAAGGGTGATTTTCACACTTCACATTTGGCGTTAGGGCTAGTATTTCACAAACATTACCGTCTGGAACTTTTGAAGGCTGAGTTAAATGACTTCATCTTGTGACAGTGCCTGACTCCCAGTAGGTGCTCAGCAAATGCTCCCATCCATCTGGGAGTATAGACTTAGGGTTTATCTATTTTTTTTTTTTTTTGGCTCTCTGGACTTTAAAACTCAGCATCTTCTGAACCAGAGGCATTTCTGATTAGCCCTTCCCTACCTATTTTCCTAGTATCACTCTTTAATCAGCTTGGGGAGGTGGCAGCATTTCATGGCCTCCGTAGTAACTCACAATGCTTCCTGGGGTATTTAAATTCTACTCTCTCATCAGCACTGAGCACCTACTTTGGGCCCTTTCCCGTGCTA

MIAT-m：CACTGAGAAGCATCTTTGCAGATAAGTATTTAAACTTACCAGCCCATAGCCTACCAGCCTGCCCTCTTCTGGTCTGTGCAGGAAAGTGTAGTATCCTAGAATGCCAAAGTGGGAGGGGAAATGGGTGATGTAGCTCATTCTCTTTTATCCATATATGGAAGAAAGAAAGAGCCTGTCCCCATTTTGTGGGGTCCCAGAAAGGGTGATTTTCACACTTCACATTTGGCGTTAGGGCTAGTATTTCACAAACATTACCGTCTGGAACTTTTGAAGGCTGAGTTAAATGACTTCATCTTGTGACAGTGCCTGACTCCCAGTAGGTGCTCAGCAAATGCTCCCATCCATCTGGGAGTATAGACTTAGGGTTTATCTATTTTTTTTTTTTTTTGGCTCTCTGGACTTTAAAACTCAGCATCTTCTGAACCAGAGGCATTTCTGATTAGCCCTTCCATCCATATTTTCCTAGTATCACTCTTTAATCAGCTTGGGGAGGTGGCAGCATTTCATGGCCTCCGTAGTAACTCACAATGCTTCCTGGGGTATTTAAATTCTACTCTCTCATCAGCACTGAGCACCTACTTTGGGCCCTTTCCCGTGCTA

let-7i-5p: TGAGGTAGTAGTTTGTGCTGTT


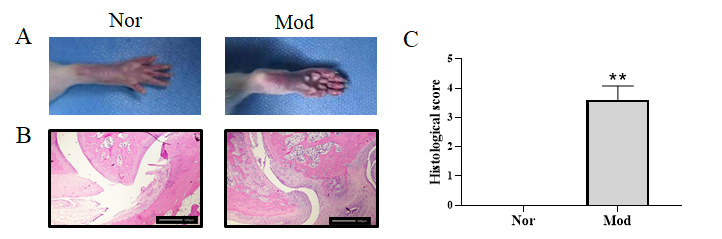


Supplementary figure 1 The establishment of CIA rat model.

(A) Representative photographs of ankle joints from each group. (B) Representative images of H&E staining. (C) Histological score.


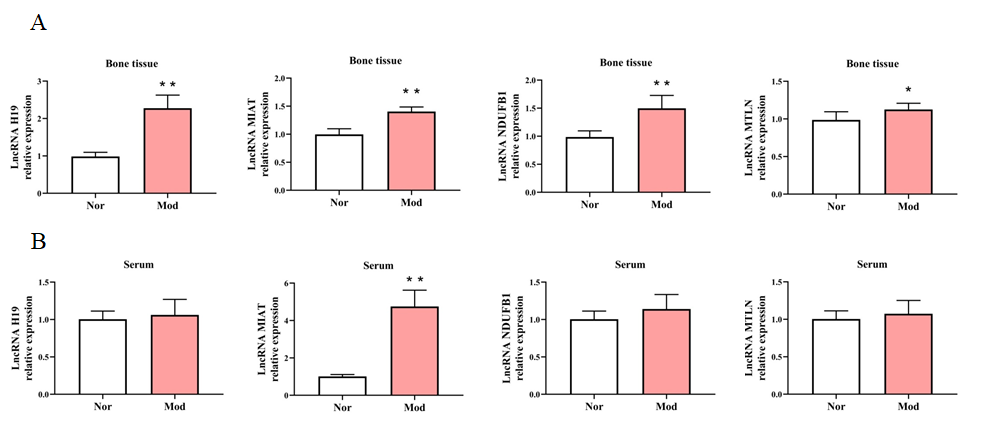


Supplementary Figure 2 Analysis the levels of lncRNAs in CIA rats.

(1) RT-qPCR analysis of lncRNAs levels in tarsal joint tissue of CIA rats. (2) RT-qPCR analysis of lncRNAs levels in serum of CIA rats.
